# Dietary diversity is associated with nutrient adequacy, blood biomarkers and anthropometric status among preschool children in poor ethnic minority area of Northwest China

**DOI:** 10.3389/fnut.2022.948555

**Published:** 2022-11-24

**Authors:** Binshuo Hu, Shihua Tang, Zhuo Wang, Youhai Chen, Xiaohui Chen, Qian Zhao, Yu Jiang, Minghui Shen, Chong Zhang, Catherine Kaliszewski, Liang Wang, Ying Zhang

**Affiliations:** ^1^School of Public Health, Lanzhou University, Lanzhou, China; ^2^Lanzhou University Second Hospital, Lanzhou, China; ^3^Gansu Provincial Maternity and Child-care Hospital, Lanzhou, China; ^4^Department of Public Health, Robbins College of Health and Human Sciences, Baylor University, Waco, TX, United States

**Keywords:** preschool children, dietary diversity, nutrient adequacy, blood biomarkers, anthropometric status

## Abstract

**Introduction:**

This study aimed to evaluate the status of dietary diversity, nutrient adequacy, blood biomarkers of nutrients, and anthropometric status, as well as to determine the predictors of dietary diversity score (DDS) and mean adequacy ratio (MAR) among preschool children in poor ethnic minority areas of northwest China.

**Methods:**

A total of 578 healthy preschool children aged 3–6 from 17 kindergartens were selected to assess dietary intake, anthropometric status (height-for-age z-score (HAZ), weight-for-age z-score (WAZ), weight-for-height z-score (WHZ), and blood biomarkers. DDS and nutrient adequacy ratio (NAR) were adopted to assess dietary diversity and micronutrient adequacy, respectively.

**Results:**

The mean DDS (ranging from 1 to 9) was relatively low (4.67 ± 1.56). Most participants consumed starchy staples, but few participants consumed organ meat. DDS was associated with serum potassium, serum iron, WAZ, HAZ, all NARs, and MAR (all *p* < 0.05). Living in urban areas (β = 0.158), higher household wealth (β = 0.116), and more caregivers’ nutritional knowledge (β = 0.022) were positively associated with DDS (all *p* < 0.01), while living in urban areas (β = 0.031), higher education of caregivers (β = 0.0.027), and higher DDS (β = 0.049) were positively associated with MAR (all *p* < 0.01).

**Discussion:**

In conclusion, dietary diversity was associated with nutrient adequacy and other health outcomes. Nutritional education and poverty alleviation are integral to improving the nutritional status of preschool children.

## Introduction

Dietary diversity score (DDS) is an indicator to evaluate whether the respondents have a diverse diet, which is defined as the number of different food groups consumed during a reference period (1). It is a widely used tool to assess dietary quality rapidly, especially nutrition and food security, because investigators can collect enough information to calculate it with only a brief questionnaire (2). Inadequate dietary diversity is a global problem, and is a prevalent concern among low-income individuals and families (3). In addition, the lack of dietary diversity is one of the leading causes of malnutrition in preschool children (4).

Preschool children are in a critical period of growth, cognitive, social, and psychological development creating a high demand for various nutrients in their life (5, 6). However, malnutrition and dietary imbalance in the preschool period will not only cause severe growth problems but also adversely affect their health in adulthood, such as intellectual disability and increased risk of chronic diseases (7, 8). Chinese children’s nutritional status has greatly improved in the past two decades. But like many developing countries, China is also facing dual challenges of overnutrition and undernutrition (9, 10). According to the China Health and Nutrition Survey 2010–2013, the prevalence of stunting was 8.1% nationwide and 11.3% in rural areas for children aged 0–5 years (11). Preschool children’s dietary pattern in some poor ethnic minority areas of northwest China differs from that in other areas, such as Linxia County and Gansu Province. The nutritional status of preschool children was worse than the national level in this area (12). For a long time, the government has issued many policies and made many positive attempts to improve children’s dietary intake. However, currently there is no corresponding nutrition improvement plan specifically for preschool children aged 3–6 years (13, 14). Additionally, China’s inherent urban-rural gap of economy and education, which makes families in rural low-income areas and ethnic minority areas cannot obtain enough diversified food, has led to the nutritional problems of preschool children (15, 16).

Many studies have shown that dietary diversity has beneficial effects on nutritional status at different ages, especially in children (3, 17–19). For instance, dietary diversity has been proven to be related to micronutrient adequacy in multiple age groups in both developing and developed countries (1, 2, 20–23). Some other studies have shown significant correlations between dietary diversity and anthropometric status [e.g., height-for-age z-score (HAZ) and weight-for-age z-score (WAZ)] or blood biomarkers (e.g., Plasma vitamin B12 and folate) in children (20, 24). In northwest China, it is difficult to carry out nutritional surveys among preschool children for many reasons, such as the underdevelopment of the economy and the lack of scientific researchers in the region. Additionally, the caregivers of preschool children are more traditional and unwilling to cooperate with completing the survey (for example, they believe that blood collection will hurt their children), which can also present difficulties in the research process. Therefore, as an intuitive and food-based indicator, maybe it is a good way to evaluate preschool children’s nutritional status. However, research on dietary diversity among preschool children in China has primarily been conducted in the eastern and central regions (19, 25), and few studies have been conducted in the northwest region, where preschool children historically have a poorer nutritional status. Additionally, DDS is influenced by various sociodemographic factors and the correlation between DDS and nutrient adequacy in poor ethnic minority areas of northwest China has not been verified yet; thus, confirming this association could be helpful to simplify the dietary surveys from complicated 24-h dietary recalls to only grasp the food groups they consumed (26). Therefore, this study aimed to (1) evaluate the status of dietary diversity, nutrient adequacy, blood biomarkers of nutrients, and anthropometric status among preschool children in poor ethnic minority areas of northwest China, (2) examine the association between dietary diversity and nutrient adequacy or other health outcomes; and (3) determine the predictors of dietary diversity and nutrient adequacy, especially local-specific predictors. It could provide some theoretical support for nutrition intervention, nutritional education, and poverty alleviation as integral aspects to improve the nutritional status of preschool children.

## Materials and methods

### Study design and study population

We used baseline data from the GSPNIP study, a preschool nutrition improvement pilot program launched by the Gansu Provincial government and the World Food Program in November 2020 in Linxia County, Gansu Province in northwest China, which aimed to improve the nutritional status of preschool children by supplying breakfast to them. Linxia County is a region with the most underdeveloped economy and complex ethnic minority situation in China. The majority of ethnic minorities were Hui and Dongxiang and observe Islam as their primary religion. The enrollment number of preschool children in the kindergartens was 758. Preschool children aged 3–6 and their caregivers agreed to participate in this study and were included in this cross-sectional study. However, the following participants were excluded from the cohort study: (1) No history of acute or chronic illness (cancer, kidney disease, liver disease, etc.). (2) No gastrointestinal symptoms (constipation, diarrhea, etc.) and fever symptoms in the past three months. (3) No infectious diseases (AIDS, hepatitis B, syphilis, etc.). (4) No history of intake of antibiotics, antiviral, antifungal, or analgesic drugs in the past three months. Caregivers of preschool children were invited to the classroom for a questionnaire (mainly includes sociodemographic information and 24-h dietary recall) while the child(ren) had an anthropometric measurement and blood collection in another classroom. To ensure that the investigators can understand the dialects of the participants, the government and kindergarten staff assisted in the translation and explanation of the responses for the investigators. Since the government and kindergartens introduced our study to the caregivers before the research began, almost all of the caregivers were willing to take their children to participate in this study. In this way, we could easily obtain the written informed consent from children’s legal guardians. The study was approved by the Medical Ethics Committee of School of Public Health, Lanzhou University, China (No. GW-20200910-1) and registered on the WHO International Clinical Trials Registry Platform (protocol code ChiCTR2200056916).

We used cluster sampling to select a representative sample of kindergartens from a sample pool of 150 kindergartens. A power analysis was conducted to ensure optimum power was achieved, resulting in a minimum sample size of 17. A total of 608 healthy preschool children aged 3–6 years from 17 kindergartens were randomly selected. All questionnaires were responded to with a response rate of 100%. Children who were ill (diseases mentioned in the exclusion criteria, *n* = 5) and had too many missing measurements (sociodemographic data had more than 3 missing blanks or dietary/anthropometric/blood biomarkers data was incomplete, *n* = 25) were excluded from the study, leading to the final sample size of 578 for analysis in this study with a valid questionnaire rate of 95.07%.

### Sociodemographic data

Sociodemographic data were collected by interviewing preschoolers’ caregivers with questionnaires. This questionnaire was adapted from the previous questionnaire of our group (3). The Cronbach test and the Kaiser-Meyer-Olkin (KMO) test were used for reliability and validity (Cronbach’s α = 0.735 and KMO = 0.802), respectively. Study variables included preschoolers’ sex, ethnicity, family’s current residence, education of caregivers, left-behind children, an only child, premature baby, feeding method, poor households, whether caregivers were engaged in farming, caregivers’ nutritional knowledge, and picky eating behavior. Left-behind children were defined as at least one parent who had gone out to work for more than 3 months, a common phenomenon in poor areas of China (27). Whether they were poor households was determined after government investigations, which avoided the bias caused by people in the region who generally like to conceal their income. Feeding methods included breastfeeding, artificial feeding, and mixed feeding, while caregivers who answered 9 questions correctly (60%) and above were considered to pass the nutritional knowledge test. Picky eating behavior was evaluated by the caregivers themselves.

### Dietary data

Throughout the study, we collected dietary intakes using face-to-face 24-h dietary recall. Participants were asked to report all foods and beverages they had consumed during the preceding 24 h. Considering many children had meals in kindergarten, we used two questionnaires to collect dietary information. One questionnaire was completed with the help of kindergartens chefs to collect information related to children’s food consumption at schools. The other questionnaire was conducted by investigators interviewing the caregivers of preschool children and aimed to collect information on what the children ate away from kindergarten. Trained investigators from Lanzhou University were responsible for collecting information on the recipes, types, and brands of all reported food items. During the interview, standard serving bowls, plates, and glasses were used to help respondents estimate the portion sizes of foods and beverages as accurately as possible. More than 50 food models for foods commonly consumed by local people were provided to help clarify the dietary intakes. Additionally, a food photo book containing photos of 135 common food and beverage items, marked with their name and its weight, was used to improve dietary recall. Nutrient analysis software (Nutrition Calculator version 2.8.0.8, Beijing, China), based on the continuously updated in-house nutrient database (China food composition), was used to calculate daily nutrient intakes and food weights from 24 h recall. Currently, the database contains information on energy and 36 nutrients for 2,876 entries. Values of energy intake and nutrient intake were used in the analysis.

### Measurement of dietary diversity and nutrient adequacy

Dietary diversity score was used to evaluate children’s dietary diversity based on the consumption of nine food groups [starchy staples (comprised of cereals and white roots and tubers); dark green leafy vegetables; other vitamin A-rich fruits and vegetables (comprised of vitamin A-rich vegetables and tubers and vitamin A rich fruit); other fruits and vegetables; organ meat; meat and fish; eggs; legumes, nuts, and seeds; milk and milk products] in the 24-h dietary recall, which was according to the guidelines of the Food and Agriculture Organization of the United Nations (28). Each food group consumed was scored 1 point and DDS was the sum of the scores (ranging from 1 to 9). The scoring did not include other food groups such as beverages, sugars, and preserves. In this study, DDS < 5 was defined as low DDS, and DDS ≥ 5 was defined as high DDS (20).

Nutrient adequacy ratios for the intake of energy, calcium, potassium, sodium, magnesium, iron, zinc, phosphorus, selenium, vitamin A, vitamin B_1_, vitamin B_2_, vitamin C, vitamin D, vitamin E, and niacin were calculated by dividing the daily intake of the nutrient by the recommended daily intake (EAR) for that nutrient according to the Chinese Dietary Reference Intakes (DRIs) (29). Because adequate intake of one nutrient cannot compensate for the lack of other nutrients, the maximum value of NAR was set to 1. MAR was used to assess the average condition of children’s nutrient adequacy and was calculated by dividing the sum of all micronutrient NARs by the quantities of nutrients evaluated.

### Anthropometric status and blood biomarkers

Height was measured to the nearest 0.1 cm using a height and sitting height measuring instrument (model SZ-200, Suheng, Shanghai, China). In contrast, weight was measured to the nearest 0.1 kg using a digital scale (model HD382, TANITA, Tokyo, Japan). WHO Anthro and WHO AnthroPlus, which the WHO officially recommended, were used to calculate the anthropometric status [HAZ, WAZ, weight-for-height z-score (WHZ)] of preschool children under 5 years old and over 5 years old, respectively. Based on this, the prevalence of stunting, wasting, and being underweight were calculated to understand the undernutrition status of preschool children (30).

To measure blood routine, 200 μl of the child’s peripheral blood sample was collected into EDTA vacutainers by automated hematology analyzer (model XS-500i, SYSMEX, Tokyo, Japan) and 500μL of the child’s peripheral blood sample was collected into lithium heparin evacuated blood collection tube to measure blood elements by atomic absorption spectrometer (model BH7100S, Bohui, Beijing, China). Hemoglobin (HGB), calcium, potassium, sodium, magnesium, iron, and zinc were measured in this study.

The following cutoffs were applied to derive binary outcomes:

• Stunting, wasting, and underweight: HAZ < −2, WHZ < −2, and WAZ < −2, respectively.
• Anemia: HGB <118 g/L (children younger than 5 years old) and HGB < 123 g/L (children aged 5 years and older) based on the standards of Gansu Provincial Maternity and Child-care Hospital (altitude-adjusted).
• Blood trace elements deficiency: Low serum calcium (<1.5 μmol/L), low serum potassium (<30 μmol/L), low serum sodium (<64 μmol/L), low serum magnesium (<1.12 μmol/L), low serum iron (<7.5 μmol/L), low serum zinc (<55.9 μmol/L) based on the standards of Gansu Provincial Maternity and Child-care Hospital.

### Statistical analysis

The database was established with EpiData version 3.1 (EpiData Association, Odense, Denmark). Descriptive statistics were taken to count the basic information of the participants. The Shapiro–Wilk test was conducted to verify the normality of the distribution of data. For normally distributed data, they were presented as mean and standard deviation (SD); For non-normally distributed data, they were presented as medians (25th and 75th percentile). To analyze the difference in DDS among different sociodemographic factors, Student’s *t*-test and one-way ANOVA were used for two-group comparisons and multiple comparisons, respectively. Differences in other variables between the high DDS and low DDS groups were tested using Student’s t-test for normally distributed continuous variables and Mann–Whitney U-test for not normally distributed continuous variables, respectively. Furthermore, to measure the correlation between DDS and other variables, Spearman’s correlation coefficients were used. Finally, Poisson regression was used to determine the predictors of DDS since it was the count data; and multiple linear regression was used to determine the predictors of MAR since it was the continuous data. Statistical significance was determined at *p* < 0.05 (two-tailed tests). All analyses were conducted with IBM SPSS (predictive analytics software and solutions) version 22.0 (International Business Machines Corporation, New York, State of New York, USA).

## Results

### Dietary diversity score based on basic characteristics

A total of 578 children (about 46.89% are ethnic minorities) aged 3–6 years old were included for analysis with a response rate of 100% and a valid questionnaire rate of 95.07% and the mean DDS was 4.67 ± 1.56. In this study, 62.28% of the caregivers had education at primary school or below, and 89.62% of them did not pass the nutritional knowledge test, while nearly two-thirds of the children were left-behind children and 79.41% of them were not an only child. Preschool children who lived in rural areas, whose parents had lower education, who were left-behind children, whose households were poor, whose caregivers’ nutritional knowledge was lacking, who were anemic, and who had low serum iron had significantly lower DDS (*p* < 0.05). However, children who had ever been a premature baby had a higher DDS (Table 1).

**TABLE 1:** The dietary diversity score (DDS) and participants’ basic characteristics (*n* = 578).

Basic characteristics	*N* (%)	DDS (Mean ± SD)	*p*
Total	578 (100)	4.67 ± 1.56	
**Gender**			
Male	321 (55.54)	4.63 ± 1.55	0.422
Female	257 (44.46)	4.73 ± 1.58	
**Ethnicity**			
Han	307 (53.11)	4.68 ± 1.39	0.857
Non-Han	271 (46.89)	4.66 ± 1.74	
**Family’s current residence**			
Rural	317 (54.84)	4.31 ± 1.49	<0.001***
Urban	261 (45.16)	5.11 ± 1.54	
**Education of caregivers**			
Primary school and below	360 (62.28)	4.39 ± 1.56^a^	<0.001***
Junior high school	150 (25.95)	5.12 ± 1.43^b^	
High school	45 (7.79)	4.98 ± 1.55^b^	
Bachelor degree and above	23 (3.98)	5.52 ± 1.56^b^	
**Left behind children**			
Yes	359 (62.11)	4.47 ± 1.54	<0.001***
No	219 (37.89)	5.00 ± 1.55	
**Only child**			
Yes	119 (20.59)	4.61 ± 1.45	0.575
No	459 (79.41)	4.69 ± 1.59	
**Premature baby**			
Yes	26 (4.50)	5.27 ± 1.37	0.032*
No	552 (95.50)	4.64 ± 1.57	
**Feeding methods**			
Breast milk	248 (42.91)	4.83 ± 1.58	0.109
Artificial feeding	153 (26.47)	4.54 ± 1.57	
Mixed feeding	177 (30.62)	4.56 ± 1.53	
**Poor households**			
Yes	199 (34.43)	4.18 ± 1.48	<0.001***
No	379 (65.57)	4.93 ± 1.55	
**Engaged in farming**			
Yes	380 (65.74)	4.45 ± 1.52	<0.001***
No	198 (34.26)	5.12 ± 1.57	
**Caregivers’ nutritional knowledge**			
Pass	60 (10.38)	5.23 ± 1.65	0.003**
Failed	518 (89.62)	4.61 ± 1.54	
**Picky eating behavior**			
Yes	325(56.23)	4.67 ± 1.46	0.988
No	253(43.77)	4.67 ± 1.69	
**Stunting**			
Yes	39(6.75)	4.23 ± 1.56	0.067
No	539(93.25)	4.71 ± 1.56	
**Wasting**			
Yes	24(4.15)	4.46 ± 1.32	0.492
No	554(95.85)	4.68 ± 1.57	
**Underweight**			
Yes	40(6.92)	4.25 ± 1.41	0.076
No	538(93.08)	4.70 ± 1.57	
**Anemia**			
Yes	33(5.71)	4.00 ± 1.67	0.011*
No	545(94.29)	4.71 ± 1.5s	
**Low serum zinc**			
Yes	73(12.63)	4.79 ± 1.51	0.478
No	505(87.37)	4.66 ± 1.57	
**Low serum calcium**			
Yes	129(22.32)	4.72 ± 1.67	0.693
No	449(77.68)	4.66 ± 1.53	
**Low serum iron**			
Yes	90(15.57)	4.20 ± 1.62	0.002**
No	488(84.43)	4.76 ± 1.54	

DDS, dietary diversity score; SD, standard deviation. ^a, b^Different superscript letters suggested a significant difference between the groups. *p* value was calculated using Student’s *t*-test or one-way ANOVA. *p < 0.05, **p < 0.01, ***p < 0.001.

### Consumption of each food group

**FIGURE 1:**
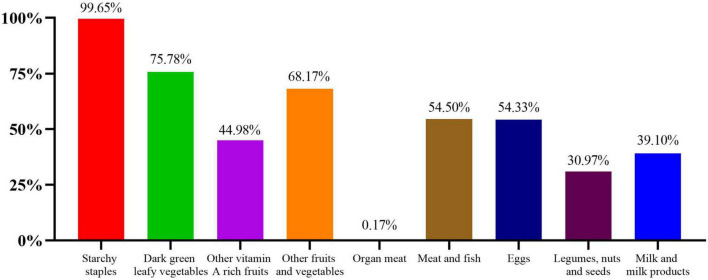
Percentage of participants consuming each food group.

The most frequently consumed food groups were starchy staples (99.65%), followed by dark green leafy vegetables (75.78%), other fruits and vegetables (68.17%), meat and fish (54.50%), and eggs (54.33%). In comparison, the groups of other vitamin A-rich fruits and vegetables (44.98%); milk and milk products (39.10%); legumes, nuts, and seeds (30.97%) were less likely to be consumed. The least consumed food group was organ meat, with only 0.17% of participants consumed (Figure 1).

**FIGURE 2:**
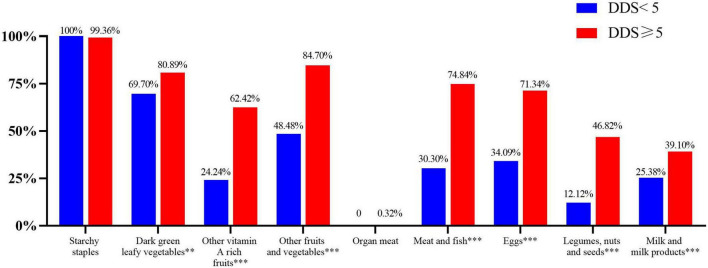
Percentage of participants consuming each food group in low and high DDS groups. **p < 0.01, ***p < 0.001.

With the exception of starchy staples and organ meat, the percentage of participants consuming each food group in the high DDS group was significantly higher than the low DDS groups (*p* < 0.05) (Figure 2).

### Blood biomarkers, anthropometric measures, and nutrient adequacy ratios in low and high dietary diversity score groups

Blood biomarkers and anthropometric status in low and high DDS groups are shown in Table 2. Serum potassium, serum iron, WAZ, and HAZ were higher in high-DDS participants than those in low-DDS participants (*p* < 0.05). Spearman correlation coefficients for blood biomarkers and anthropometric status associated with DDS were determined for all participants. Serum potassium (*r* = 0.201, *p* = 0.001), serum iron (*r* = 0.208, *p* = 0.001), WAZ (*r* = 0.098, *p* = 0.019) and HAZ (*r* = 0.097, *p* = 0.020) were significantly positively correlated with DDS.

**TABLE 2:** Blood biomarkers and anthropometric status in low and high DDS groups.

	DDS < 5	DDS ≥ 5	*p* ^a^	*r* ^b^	*p*
HGB	141.00 (133.75, 147.00)	141.50 (136.00, 148.00)	0.097	0.076	0.067
Serum calcium	1.60 ± 0.14	1.59 ± 0.13	0.671	–0.008	0.845
Serum potassium	41.27 ± 3.07	42.08 ± 3.43	0.003**	0.201	< 0.001***
Serum sodium	75.55 ± 5.92	75.77 ± 5.42	0.628	0.042	0.314
Serum magnesium	1.48 ± 0.13	1.49 ± 0.14	0.533	0.074	0.077
Serum iron	8.09 ± 0.74	8.29 ± 0.70	< 0.001***	0.208	< 0.001***
Serum zinc	67.65 ± 10.66	68.96 ± 11.04	0.151	0.061	0.145
WHZ	−0.59 (−1.26, 0.08)	−0.48 (−1.11, 0.09)	0.257	0.044	0.295
WAZ	−0.74 ± 0.96	−0.55 ± 0.90	0.020*	0.098	0.019*
HAZ	−0.57 ± 1.02	−0.36 ± 1.05	0.015*	0.097	0.020*

Values are mean ± SD or medians (25th and 75th percentile). HGB, hemoglobin; WHZ, weight-for-height z-score; WAZ, weight-for-age z-score; HAZ, height-for-age z-score. ^a^*p*-Values were calculated by Mann–Whitney U-test for non-normally distributed continuous variables and Student’s t-test for normally distributed continuous variables. ^b^Spearman’s correlation coefficients (*r*) were calculated between participants’ DDS and blood biomarkers or anthropometric status. *p < 0.05, **p < 0.01, ***p < 0.001.

**FIGURE 3:**
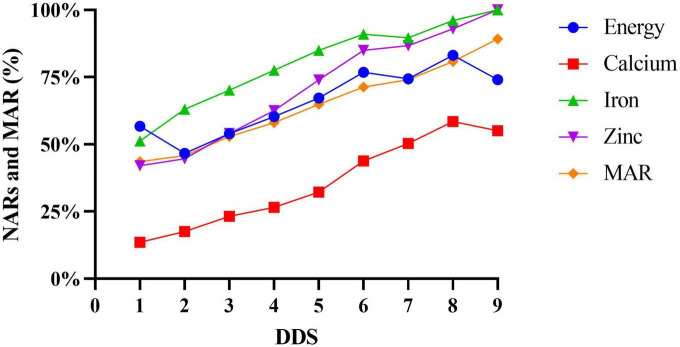
NARs and MAR at different levels of dietary diversity score.

**FIGURE 4:**
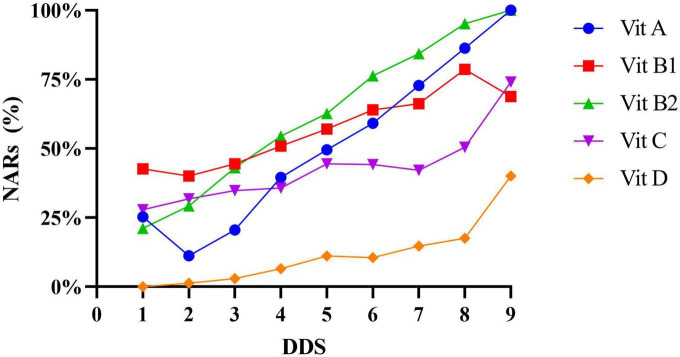
NARs of selected vitamins at different levels of dietary diversity score.

In the same way, of the 15 micronutrients NAR values assessed, all of them and MAR were higher in the high-DDS participants than in the low-DDS participants (*p* < 0.01). Moreover, energy and macronutrients NAR values were significantly higher in the high DDS group than those in the low DDS group (*p* < 0.01). In the correlation study, the NARs of all micronutrients and MAR were significantly positively correlated with DDS (*r* value range = 0.124–0.593, *p* < 0.001). Similar results also occurred in energy and macronutrients NAR values (Table 3). In this study, the relationship between DDS and the NARs of energy, selected minerals, and vitamins showed an increase in NARs for all these nutrients as DDS increased (Figures 3, 4). When DDS reached its maximum value of 9, NARs of iron, zinc, vitamin A and vitamin B2 reached 100%. However, NARs of the other nutrients did not meet 100% whatever the DDS was. In addition, the most common nutrients were calcium and vitamin D, which were only 40% at the highest.

**TABLE 3:** NARs and MAR in low and high DDS groups.

NARs	DDS < 5	DDS ≥ 5	*p* ^a^	*r* ^b^	*p*
Energy	0.56 ± 0.21	0.72 ± 0.21	< 0.001***	0.439	< 0.001***
Protein	0.74 ± 0.31	1.07 ± 0.36	< 0.001***	0.549	< 0.001***
Fat	0.41 (0.26, 0.60)	0.64 (0.50, 0.81)	< 0.001***	0.524	< 0.001***
Carbohydrate	0.67 ± 0.27	0.79 ± 0.27	< 0.001***	0.247	< 0.001***
Calcium	0.17 (0.11, 0.31)	0.32 (0.19, 0.54)	< 0.001***	0.511	< 0.001***
Potassium	0.54 ± 0.21	0.73 ± 0.21	< 0.001***	0.456	< 0.001***
Sodium	0.94 ± 0.20	0.98 ± 0.12	0.004**	0.124	0.003**
Magnesium	0.66 ± 0.22	0.82 ± 0.17	< 0.001***	0.429	< 0.001***
Iron	0.72 ± 0.23	0.88 ± 0.16	< 0.001***	0.421	< 0.001***
Zinc	0.57 ± 0.23	0.80 ± 0.20	< 0.001***	0.561	< 0.001***
Phosphorus	0.89 ± 0.19	0.93 ± 0.15	0.009**	0.157	< 0.001***
Selenium	0.71 ± 0.26	0.77 ± 0.25	0.005**	0.213	< 0.001***
Vitamin A	0.19 (0.05, 0.43)	0.55 (0.34, 0.88)	< 0.001***	0.587	< 0.001***
Vitamin B_1_	0.43 (0.33, 0.59)	0.60 (0.45, 0.77)	< 0.001***	0.415	< 0.001***
Vitamin B_2_	0.39 (0.23, 0.68)	0.74 (0.49, 1.00)	< 0.001***	0.550	< 0.001***
Vitamin C	0.29 (0.18, 0.46)	0.37 (0.23, 0.60)	< 0.001***	0.202	< 0.001***
Vitamin D	0.00 (0.00, 0.06)	0.10 (0.04, 0.16)	< 0.001***	0.494	< 0.001***
Vitamin E	0.78 ± 0.27	0.94 ± 0.14	< 0.001***	0.387	< 0.001***
Niacin	0.41 (0.28, 0.59)	0.65 (0.47, 0.82)	< 0.001***	0.428	< 0.001***
MAR	0.54 ± 0.14	0.69 ± 0.12	< 0.001***	0.593	< 0.001***

Values are mean ± SD or medians (25th and 75th percentile). NAR, nutrient adequacy ratio; MAR, mean adequacy ratio. ^a^*p*-values were calculated by Mann–Whitney U-test for non-normally distributed continuous variables and Student’s *t*-test for normally distributed continuous variables. ^b^Spearman’s correlation coefficients (*r*) were calculated between participants’ DDS and NARs. **p < 0.01, ***p < 0.001.

### Predictors of dietary diversity score and mean adequacy ratio

In the Poisson regression model (Table 4), living in urban areas [β = 0.158, 95%*CI*: (0.075, 0.241)], higher household wealth [β = 0.116, 95%*CI*: (0.030, 0.202)], and more caregivers’ nutritional knowledge [β = 0.022, 95%*CI*: (0.006, 0.038)] were positively associated with DDS. Similarly, in the multivariable linear regression models (Table 5), being female [β = 0.025, 95%*CI*: (0.006, 0.043)], living in urban areas [β = 0.031, 95%*CI*: (0.010, 0.051)], higher education of caregivers [β = 0.027, 95%*CI*: (0.013, 0.040)] and higher DDS [β = 0.049, 95%*CI*: (0.042, 0.055)] were positively associated with MAR, while preschool children’s age [β = −0.057, 95%*CI*: (−0.078, −0.035)] and being non-Han Chinese [β = 0.030, 95%*CI*: (−0.050, −0.010)] were negatively associated with MAR.

**TABLE 4:** Poisson regression model of predictors of DDS.

Variables	β	95%CI	*p*
Age	–0.066	(−0.155, 0.023)	0.148
Sex	0.022	(−.055, 0.098)	0.581
Female (vs. male)			
Ethnicity	0.058	(−0.023, 0.139)	0.160
Non-Han (vs. Han)			
Family’s current residence	0.158	(0.075, 0.241)	< 0.001***
Urban (vs. rural)			
Education of caregivers^a^	–0.008	(−0.061, 0.045)	0.760
Left behind children	0.044	(−0.038, 0.126)	0.291
No (vs. yes)			
Only child	0.023	(−0.071, 0.117)	0.628
No (vs. yes)			
Premature baby	–0.088	(−0.251, 0.075)	0.292
No (vs. yes)			
Poor households	0.116	(0.030, 0.202)	0.008**
No (vs. yes)			
Engaged in farming	0.056	(−0.027, 0.139)	0.184
No (vs. yes)			
Caregivers’ nutritional knowledge^b^	0.022	(0.006, 0.038)	0.006**

^a^Primary school and below, junior high school, high school, bachelor degree and above (education of caregivers) were assigned as 1, 2, 3, 4 respectively. ^b^Caregivers’ nutritional knowledge was analyzed using continuous variable nutritional knowledge score. **p < 0.01, ***p < 0.001.

**TABLE 5:** Linear regression model of predictors of MAR.

Variables	β	95%CI	*p*
Age	–0.057	(−0.078, −0.035)	< 0.001***
Sex	0.025	(0.006, 0.043)	0.010*
Female (vs. male)			
Ethnicity	–0.030	(−0.050, −0.010)	0.003**
Non-Han (vs. Han)			
Family’s current residence	0.031	(0.010, 0.051)	0.004**
Urban (vs. rural)			
Education of caregivers^a^	0.027	(0.013, 0.040)	< 0.001***
Left behind children	0.005	(−0.015, 0.025)	0.647
No (vs. yes)			
Only child	–0.020	(−0.043, 0.001)	0.056
No (vs. yes)			
Premature baby	–0.014	(−0.045, 0.017)	0.380
No (vs. yes)			
Poor households	0.012	(−0.008, 0.033)	0.245
No (vs. yes)			
Engaged in farming	0.006	(−0.013, 0.025)	0.555
No (vs. yes)			
Caregivers’ nutritional knowledge^b^	0.002	(−0.002, 0.006)	0.247
DDS	0.049	(0.042, 0.055)	< 0.001***

^a^Primary school and below, junior high school, high school, bachelor degree and above (education of caregivers) were assigned as 1, 2, 3, 4, respectively. ^b^Caregivers’ nutritional knowledge was analyzed using continuous variable nutritional knowledge score. *p < 0.05, **p < 0.01, ***p < 0.001.

## Discussion

This study assessed the status of anthropometric status, blood biomarkers of nutrients, nutrient adequacy, and dietary diversity, as well as their relationships and the possible influencing factors of DDS and NAR among preschool children in poor ethnic minority areas of northwest China. The present study demonstrated that dietary diversity was positively associated with all nutrients (both macro- and micro- nutrients) and several other health outcomes.

The results showed that the mean DDS of preschool children was 4.67 ± 1.56, which was lower than the DDS reported in other areas of Chinese studies [5.77 in Chen et al., 6.10 in Meng et al., 6.80 in Zhao et al., 7.4 in Jiang et al. (19, 25, 31, 32)]. Compared to other developing countries, their DDS was higher than the preschool children in South Africa [3.60 in Steyn et al. (33)] and Zambia [4.39 in Caswell et al. (2)] but lower than those in the Philippines [5.62 in Modjadji et al. (34)] and Sri Lanka [5.4 in Perkins et al. (24)]. DDS has been used for many years, but the scoring standard of DDS is still not uniform. Different food groupings, 1-day or 3-day dietary recall, and whether there was a threshold of food intake when calculating DDS all affected the DDS value. Similarly, the prevalence of stunting, underweight, and wasting were 6.75, 6.92, and 4.15%, respectively, which were higher than in other studies conducted in Guangdong Province [1.31, 1.03, and 2.06% in Mu et al. (35)] or Shaanxi Province [1.24, 0.06, and 0.07% in Ding et al. (36)]. One possible explanation is that previous studies have concentrated on the central or eastern regions, where people have better access to higher socioeconomic status and nutritional concepts, which may contribute to the observed gap between this study and other studies. Additionally, the prevalence of low serum zinc, calcium, and iron were 12.63, 22.32, and 15.57%, respectively. Conversely, the prevalence of anemia was only 5.71%. The results of this study are generally similar to other studies, the iron deficiency rate is higher than that of Zhejiang Province (one of the most economically developed provinces in southeast China) but lower than that of Qinghai Province (in northwest China) (37, 38). Furthermore, the prevalence of anemia is quite low compared to other studies [19% in Wang et al., 11.19% in Zeng (39, 40)]. As of 2020, the “Nutrition Package” program of “1,000 days in early life” had been implemented in impoverished areas in China for many years. With the help of the “Nutrition Package,” Chinese preschool children could supplement more micronutrients in their early life, as one of the reasons for the low prevalence of anemia in local children (13).

We also found that participants with key different characteristics had varying DDS. Specifically, participants who lived in rural areas had lower DDS than those living in urban areas, which was consistent with many studies (18, 19, 24, 31, 32). This is probably due to reduced access to diverse foods and can be more costly compared to individuals living in urban areas (18, 41). Studies also reported that participants with caregivers’ that had a higher education level and family economic status in turn, had a higher DDS (18, 26, 42–44). Our present study also supports this finding. Left-behind children were a common phenomenon in impoverished areas in China, where at least one parent had gone out to work for more than 3 months. This may lead to children’s lack of dietary diversity, as evidenced by the findings of this study and other similar studies (45). Additionally, we also found that caregivers with more nutritional knowledge could contribute to higher DDS of preschool children, as other studies pointed out (25). In our study, participants who had anemia and low serum iron had a lower DDS, which has not been investigated in many studies. However, contrary to our hypothesis, preschool children who were born prematurely had a higher DDS. One possible explanation is that caregivers of premature babies used to give them more care and better food due to the poor condition when they were born.

Many reasons have been proposed to explain why people consume certain food groups instead of others (46). Two important reasons may include costs and accessibility (25, 38, 46). In our study, most of the food preschool children ate was planted by their own families, such as potatoes, Chinese cabbage, spinach, and tomatoes. This is the reason why the consumption of starchy staples, dark green leafy vegetables, and other fruits and vegetables were high. In addition, animal foods can provide various nutrients such as iron, calcium, and vitamin D, which cannot be replaced by plant-based foods (47). However, animal product consumption in preschool children was lower than plant-based foods consumption, especially organ meat, which was minimally consumed by anyone. Ethnic differences could explain this result, as half of the participants were ethnic minorities and Muslims, which prevented them from consuming organ meats. Thus, these foods were rare in local markets (48). After grouping according to DDS, the proportion of participants consuming all food groups except starchy staples and organ meat in the high DDS group was higher than that in low DDS group, which was also similar to other studies (23, 49).

To our knowledge, the present study is the first to assess the relationship between dietary diversity and blood biomarkers or anthropometric status among preschool children in a poor ethnic minority area of China. DDS is positively associated with serum potassium, serum iron, WAZ, and HAZ. For blood biomarkers, Vyncke et al. found that Diet Quality Index (similar to DDS) was positively associated with 25-hydroxyvitamin D, holo-transcobalamin and n-3 fatty acid serum levels in European adolescents (50), while Ganpule-Rao et al. found that DDS was positively associated with vitamin B_12_, folate, and Hb in Indian rural youth (20). For anthropometric status, only a positive association between DDS and HAZ in Sri Lankan young children was observed by Perkins et al. (24). Compared with dietary intake, blood biomarkers or anthropometric status were medium and long-term indicators that reflected the nutritional status of preschool children, so the link with DDS is not too strong. However, the results of this study suggested that DDS could also assess blood biomarkers or anthropometric status in preschool children to some extent.

Simultaneously, a positive correlation was also found between DDS and NARs in all macro- and micronutrients, which was in accordance with but much stronger than previous findings (1, 3, 19, 21, 49). Although there were very few participants with a DDS of 1 and 9 (only 4 and 1, respectively) and random errors would be introduced in studying the relationship between DDS and NARs and MAR, we can generally infer that even when the DDS was 9, part of NARs and MAR were still less than 100% (2, 18, 33, 51). However, many NARs (such as calcium and vitamin D) were quite low in both high DDS and low DDS groups, suggesting that preschool children were at risk of hidden hunger. In our study, both the number of consumers and the consumption of milk and dairy products, as well as meat and fish, were very limited, which was undoubtedly a key factor in the observed low NARs.

According to the Poisson regression model of predictors of DDS, living in urban areas, higher household wealth and more caregivers’ nutritional knowledge were positive influential factors of DDS, which was consistent with many studies (25, 32). Similarly, living in urban areas, higher education of caregivers, and higher DDS were positively associated with MAR, illustrating the positive association between DDS and NAR. Thanks to the grassroots poverty alleviation work vigorously promoted by the Chinese government, we can expect that the nutritional status of preschool children will be improved accordingly. Also, in part with the help of the constantly increasing level of economic development and living quality of individuals in poor ethnic minority areas dietary intake will be improved in the near future. In addition, nutritional education for caregivers could also be given attention to improve the nutritional status of preschool children.

China is an expansive country with diverse lifestyles and nutrition intake. To the best of our knowledge, our study is the first survey focused on the association between dietary diversity and nutrient adequacy, blood biomarkers, anthropometric status as well as exploring the predictors of DDS and MAR in preschool children in poor ethnic minority area of northwest China. The findings could help evaluate the nutritional status of preschool children via an alternative approach that could simplify the workload of dietary surveys and provide preschool children with much more balanced diets, particularly making up for the lack of using only one type of indicator (e.g., blood trace element) in such poor areas. There were also several limitations to this study. First, the study design was a cross-sectional investigation and it could not infer a causal relationship. Second, it was conducted only in one county of one province, and the data might be not representative. Third, due to the local caregivers’ traditional thinking and religious beliefs, only peripheral blood was collected instead of venous blood, which was a limitation that we could not analyze biomarkers such as plasma vitamins. Fourth, it is difficult to control some confounders such as biological variations.

## Conclusion

In conclusion, this study indicated that the dietary diversity was relatively low with starchy staples being the most consumed food group and the intakes of many nutrients were inadequate. While the prevalence of stunting, wasting, and underweight was high among preschool children in poor ethnic minority area of Northwest China. Additionally, DDS was associated with serum potassium, serum iron, WAZ, HAZ, all NARs, and MAR. Efforts are warranted to increase the dietary diversity of preschool children in such areas to improve their nutritional status. Furthermore, living in urban areas, higher household wealth, not engaging in farming, and more caregivers’ nutritional knowledge were positively associated with DDS while living in urban areas, higher education of caregivers, and higher DDS were positively associated with MAR. Nutritional education for caregivers and policies such as poverty alleviation and nutrition improvement to promote the dietary diversity of preschool children are necessary.

## Data availability statement

The raw data supporting the conclusions of this article will be made available by the authors, without undue reservation.

## Ethics statement

The studies involving human participants were reviewed and approved by the Medical Ethics Committee of School of Public Health, Lanzhou University. Written informed consent to participate in this study was provided by the participants’ legal guardian/next of kin.

## Author contributions

BH and YZ designed research. BH, ZW, ST, YC, YJ, QZ, XC, MS, and CZ conducted research. BH analyzed data and wrote the manuscript. BH and CK edited the manuscript. LW and YZ had primary responsibility for final content. All authors read and approved the final manuscript.
